# ALCOHOL CONSUMPTION AND MEMORY: A LONGITUDINAL ANALYSIS OF RECIPROCAL IMPACTS ACROSS OLD AGE

**DOI:** 10.1093/geroni/igz038.2416

**Published:** 2019-11-08

**Authors:** Oliver K Schilling

**Affiliations:** Department of Psychological Ageing Research, Institute of Psychology, Heidelberg University, Heidelberg, Germany

## Abstract

Research on the association of alcohol consumption with cognitive aging revealed mixed evidence: Whereas a u-shaped relationship has been found in many studies, suggesting that low to moderate alcohol consumption predicts more favorable cognitive outcomes than abstinence, other findings suggest that alcohol is a more linearly related risk factor for cognitive decline. These inconsistencies may partly be due to methodological variation in the statistical modeling of intraindividual changes in both, alcohol consumption and cognition across old age. The present study analyzed longitudinal change in and the mutual effects between alcohol consumption habits and verbal episodic memory (word list recall), using vector autoregressive (VAR) mixed models with nonlinear cross-lagged effects. Data from the English Longitudinal Study of Ageing was examined, including N=13388 aged 50+ (M=67.6, SD=9.25; 54.7% female), assessed at up to eight occasions with two-year follow-up intervals (2002/3–2016/17). The self-reported one-year frequency of alcohol drinking days (ADD) served as indicator of alcohol consumption. Basically, ADD predicted follow-up memory performance in a reverse u-shaped fashion, indicating best memory performance after moderate ADD, compared with both ends of the ADD continuum (i.e., drinking never vs. every day). Considering moderators, most notably age did not interact with cross-lagged effects, suggesting that those observed across an older age-range were not more (or less) vulnerable to effects of alcohol consumption on memory performance. Thus, this study adds further support for non-detrimental, if not beneficial, effects of moderate alcohol consumption on cognitive aging – regarding in particular age-related loss of episodic memory.

